# Demonstrating the Efficacy of Core-Shell Silica Catalyst in Depolymerizing Polycarbonate

**DOI:** 10.3390/polym16223209

**Published:** 2024-11-19

**Authors:** Onofrio Losito, Pasquale Pisani, Alessia De Cataldo, Cosimo Annese, Marina Clausi, Roberto Comparelli, Daniela Pinto, Lucia D’Accolti

**Affiliations:** 1 Chemistry Department, University of Bari Aldo Moro, Via E. Orabona 4, 70126 Bari, Italy; onofrio.losito@uniba.it (O.L.); p.pisani5@studenti.uniba.it (P.P.); a.decataldo3@phd.poliba.it (A.D.C.); 2 Dipartimento di Meccanica, Matematica e Management (DMMM), Politecnico di Bari, Via E. Orabona 4, 70126 Bari, Italy; 3 Dipartimento di Scienze della Vita, della Salute e delle Professioni Sanitarie, Università degli Studi Link, Via del Casale di San Pio V, 44, 00165 Roma, Italy; cosimo.annese@cnr.it; 4 CNR-ICCOM-SS, BARI (I), Via Orabona 4, 70125 Bari, Italy; 5 Dipartimento di Scienze della Terra e Geoambientali, Università degli Studi di Bari Aldo Moro, Via E. Orabona 4, 70126 Bari, Italy; marina.clausi@uniba.it (M.C.); daniela.pinto@uniba.it (D.P.); 6 CNR-IPCF-SS, BARI (I), Via Orabona 4, 70125 Bari, Italy; roberto.comparelli@cnr.it

**Keywords:** core-shell catalyst, depolymerization, polycarbonate, ionic liquids, zinc oxide, silica

## Abstract

Polycarbonate (PC) is a highly versatile plastic material that is extensively utilized across various industries due to its superior properties, including high impact strength and heat resistance. However, its durability presents significant challenges for recycling and waste management. Polycarbonate is a thermoplastic polymer representative of the class of condensation reaction polymers obtained from the reaction of bisphenol A (BPA) and a carbonyl source, such as phosgene or alkyl and aryl carbonate. The recycling processes for PC waste include mechanical recycling, blending with other materials, pyrolysis, and chemical recycling. The latter is based on the cleavage of carbonate units to their corresponding monomers or derivatives through alcoholysis and/or hydrolysis and ammonolysis, normally under basic conditions and without catalysts. This study investigates the efficacy of the use of several heterogeneous catalysts based on silica gel as a robust support, including Sc(III)silicate (thortveitite), which has been previously reported for the preparation of polyesters, core-shell Si-ILs, and core-shell Si-ILs-ZnO, which has never been used before in the depolymerization of polycarbonate, proposing a sustainable and efficient method for recycling this valuable polymer. We chose to explore core-shell catalysts because these catalysts are robust and recyclable, and have been used in very harsh industrial processes. The core-shell silica catalysts used in this study were characterized by XRD; SEM_EDX, FT-IR, and ICP-OES analysis. In our experimental protocol, polycarbonate samples were exposed to the catalyst under controlled conditions (60–150 °C, for 12–24 h) using both oxygen and nitrogen nucleophiles. The depolymerization process was systematically monitored using advanced analytical techniques (GC/MS and GPC chromatography). The experimental results indicated that core-shell silica catalyst exhibits high efficacy, with up to 75% yield for the ammonolysis reaction, producing monomers of high purity. These monomers can be reused for the synthesis of new polycarbonate materials, contributing to a more sustainable approach to polycarbonate recycling.

## 1. Introduction

**Figure 1:**
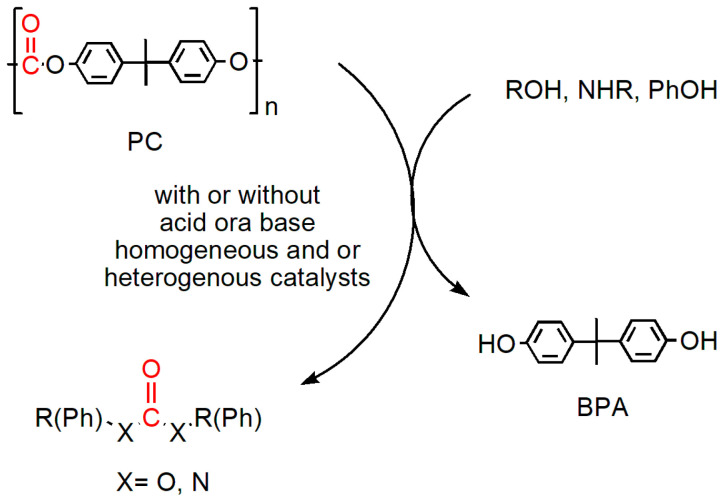
General PC depolymerization reaction.

Polycarbonate is a commonly used plastic in various industries due to its desirable properties such as high impact resistance, transparency, and heat resistance [1]. However, the disposal of polycarbonate waste presents significant environmental challenges due to its non-biodegradable nature, leading to its persistence in the environment for extended periods, potentially spanning hundreds of years. This persistence results in the generation of polycarbonate microplastics, which are frequently detected in waste-activated sludge, thereby raising further environmental concerns [2]. The overall contribution of plastic consumption to climate change is significant; by 2050, plastic production and incineration could require up to 13% of the remaining carbon budget to limit global warming to 1.5 °C [3]. Only a circular and recycling approach can solve the problem of the dichotomy between the importance of plastic and the need to reduce our environmental impact [4]. One promising approach to address this issue is the depolymerization of polycarbonate to its monomer constituents (Figure 1) using hydrolysis, ammonolysis, and alcoholysis [5,6,7,8]. These methods offer additional environmental benefits by generating non-toxic byproducts such as organic carbonates, carbamates, or urea, rather than phosgene COCl_2_, which is typically produced through the conventional pyrolysis of polycarbonate (PC).

The conventional procedures for polycarbonate (PC) depolymerization are typically conducted under high pressure and elevated temperatures, often with the employment of significant quantities of concentrated acids or bases over extended processing times [9]. Various methods have been utilized, including the use of hot compressed water, alkaline solutions, supercritical ethanol, and microwave irradiation. Metal catalysts, such as manganese(II) acetate (Mn(OAc)2) and a range of metal oxides and salts, are commonly employed [10]. Additionally, commercial manganese (II) ethyl/butyl phosphonate silica (Si-Mn) is used as a heterogeneous catalyst [11]. Recent advancements include the homogeneous depolymerization of polycarbonates utilizing a combination of zinc chloride (ZnCl2) as a Lewis acid and poly(ethylene glycol) as an additive under rigorous conditions (vacuum and temperatures exceeding 200 °C for several hours) [12].

Alternatively, waste polycarbonate can be depolymerized in the absence of additives or catalysts by utilizing basic conditions at room temperature and employing only primary and secondary amines [13]. Moreover, the methanolysis of polycarbonates catalyzed by Si-TBD (1,5,7-triazabicyclo [4.4.0]dec-5-ene) has been reported to achieve a good conversion and yield at 65 °C [8]. Otherwise, organocatalysis and, in particular, the use of tetramethylammonium methylcarbonate, have been studied for the depolymerization of various plastics, including the methanolysis of polycarbonate [14].

Ionic liquids (ILs) remain significantly underexplored in this context, with limited studies reporting the use of [BMIM][Cl] and [BMIM][OAc] as reaction media [15] under homogeneous conditions [10,16,17]. The most relevant problem in these procedures is represented by the nature of the cation, as imidazolium cations are considered poorly biodegradable and toxic [18]. It is well known that the degradation of imidazolium cations returns some cation fragments that persist in the environment [19].

To solve these drawbacks, using a procedure from the previous literature [20], some of us have made a trans polymerization by starting from commercial polycarbonate (PC) to obtain a bio-based polymer by replacing the bisphenol a (BPA) with tetrahydrocurcumin (THCM), using a circular economy approach. In this case, the depolymerization reaction was carried out with ZnO nanoparticles and tetrabutylammonium acetate as the catalytic system, making it semi-homogeneous [20]. Ionic liquids and metal salts are among the most extensively studied catalysts due to their high efficiency and minimal environmental impact. In the context of polycarbonate (PC) methanolysis, ionic liquids have demonstrated significant advantages in terms of the conversion and yield of bisphenol A (BPA). However, several challenges remain unresolved, particularly related to the complexity of the reaction work-up process and the economic viability of ionic liquids [21]. Consequently, the proposed strategy for PC depolymerization involves the development of a fully heterogeneous catalyst system comprised of two key components: (I) a non-critical metal, such as zinc, and (II) a heterogenized ionic liquid, with both being supported on mesoporous silica. The choice of mesoporous silica as a support material (due to its low cost, stability, and non-toxicity) will facilitate the efficient recyclability of the catalyst system.

Various strategies exist for immobilizing ionic liquids (ILs); however, these methods often face challenges such as material leaching and reduced catalytic activity [22]. In recent years, core-shell catalysts have garnered significant attention due to their capacity to preserve the catalytic properties of ILs through physical confinement. Traditionally, active components have been introduced into these structures post-synthesis. However, a novel approach has recently been proposed that allows for the integration of ionic liquids (ILs), specifically 1-ethyl-3-methylimidazolium bromide (EmimBr), directly during the preparation of core-shell catalysts. This is achieved by utilizing suitable quantities of IL and tetraethyl orthosilicate (TEOS) in the synthesis process [21]. In this work, we decide to explore the efficacy of a new core-shell silica structure with BMIMBr and zinc oxide (ZnO) included in the depolymerization of polycarbonate. By employing this catalyst in the depolymerization process, we aim to achieve high efficiency and selectivity in producing polycarbonate monomers. Through our experimental studies, we will demonstrate the effectiveness of core-shell silica as a catalyst in polycarbonate depolymerization and discuss the factors influencing its performance. In addition, we compared this new catalyst with a scandium silicate catalyst that we previously used in the synthesis of polyglycerol [23].

This research contributes to the development of sustainable recycling methods for polycarbonate waste, potentially mitigating the environmental impact of plastic pollution.

## 2. Materials and Methods

### 2.1. Materials

Methyl-tethrahydrofurane (m-THF); 1-butyl-3-methylimidazolium bromide (BMIMBr); hexamethylbenzene (HMB); potassium chloride (KCl); tetraethyl orthosilicate (TEOS); zinc oxide (ZnO); hydrochloric acid (HCl); Pluronic P123; scandium trifluoromethanesulfonate (Sc(OTf)3); citric acid; sodium hydroxide (NaOH); polycarbonate; bisphenol A (BPA); bisphenol A–based polycarbonate (BPA-PC; average molecular mass of 45,000 g/mol); sodium chloride (NaCl); methanol; benzylamine; dibutylamine were purchased from Sigma-Aldrich (Milan, Italy) and used as received, without any further treatment.

### 2.2. Characterization

Reaction products were identified using a Shimadzu GLC 17-A instrument (Shimadzu, Milan, Italy) equipped with an SLB-5MS column (30 m × 0.25 mm ID, film thickness 0.25 µm) by comparing with data from the literature or authentic samples. Mass spectra were acquired in EI mode (70 eV). Depolymerization percentages were determined by measuring the area of BPA and extracting the concentration from a calibration curve of BPA (Sigma-Aldrich) as described in the Supplementary Materials section.

X-ray powder diffraction (XRPD) analyses were performed using a Philips X’PERT PRO equipped with spinner and panalytical “X’Celerator” solid-state detector (CuKα radiation, Ni filter), from Irvine, CA, USA. The operating conditions were: 40 kV and 40 mA, range 3–70° 2 θ, scan speed 0.035°2 θ/min. The XRPD data were analysed using the software X’Pert High Score 3.0e (Malvern PANalytical, Almelo, the Netherlands), which includes the ICSD database.

Microscopic images were acquired using a stereomicroscope Nikon JAPAN SMZ800, from Melville, NY, USA. magnification range 10–60×. FE-SEM-EDS was performed using a (UHR-SEM) ZEISS CrossBeam 350 field emission ultra high-resolution scanning electron microscope equipped with an EDAX OCTANE Elite Plus-Silicon drift-type energy dispersive spectrometer. Images were collected using an Everhart Thornley image detector at a working distance of 11 mm with an acceleration voltage of 15 kV and probe current of 100 pA.

EDS analyses (on spots) were performed with accelerating voltage of 15 kV and working distance of 11 mm, acquiring for 30 s per spot analysis.

Transmission electron microscopy (TEM) analysis was conducted using a JEOL (Akishima City, Japan) JEM-1011 microscope operating at 100 kV. Samples were prepared by depositing a drop of a diluted NP suspension in 2-propanol onto carbon-coated copper grids. Statistical size analysis was carried out using ImageJ software (v.1.52a).

Field emission scanning electron microscopy (FE-SEM) investigation was performed with a Zeiss Sigma microscope (Carl Zeiss Co., Oberkochen, Germany) with an operating voltage range of 0.5–20 kV and equipped with an in-lens secondary electron (SE) detector, a backscattered electron (BSE) detector, and an INCA energy dispersive spectroscopy (EDS) detector. Samples were prepared by drop casting the NP dispersion in 2-propanol onto a silicon slide, which was fitted on a stainless-steel holder using double-sided carbon tape and grounded with silver paste.

ATR-FTIR spectra were obtained using a Perkin-Elmer UATR-Two spectrophotometer equipped with a single reflection diamond ATR crystal (refractive index 2.4). Spectra were acquired with 32 scans in the range of 4000–600 cm^−1^, applying both baseline and ATR corrections.

ICP-OES analyses were carried out using a Thermo Fisher iCAP 7200 Duo, after acid mineralization of both core-shell catalysts.

TGA analyses were performed using a PerkinElmer Pyris 1 TGA thermogravimetric analyzer, with both TGA and differential thermogravimetric (DTG) curves being recorded. Measurements were conducted at a heating rate of 10 °C/min from 25 °C to 600 °C under continuous nitrogen flow.

For GPC analysis, a Knauer Smartline 1000 GPC equipped with a UV detector and an Azura RI detector was used. Analyses were carried out using THF as the eluent, with UV detection in the 200–250 nm range and refractive index detection. Catalytic tests were conducted in a stainless-steel autoclave equipped with an electric oven and a magnetic stirrer. The catalyst, solvent, and reagents were introduced into a glass vial placed within the autoclave to prevent contact with the metal walls.

### 2.3. Preparation of Mesoporous Silica (KIT-6)

Mesoporous silica (KIT-6) was synthesized by dissolving 0.9 g of the surfactant Pluronic P123 (0.15 mmol, average Mn 5800), 1.11 mL of n-butanol (density = 0.81 g/mL, 12.13 mmol), and 1.43 mL of HCl (37%) in 32.55 mL of distilled water. Subsequently, 2.07 mL of tetraethyl orthosilicate (TEOS) (density = 0.933 g/mL, 9.27 mmol) was added to the solution, and the resulting suspension was stirred at 35 °C for 24 h. This mixture was then subjected to hydrothermal treatment in an autoclave at 100 °C for 24 h, followed by filtration. The resulting solid was washed, dried at 80 °C for 12 h, and calcined at 550 °C for 5 h to remove the organic template, resulting in mesoporous silica classified as KIT-6. The KIT-6 was only used for the preparation of Sc(III)silicate (thortveitite) catalyst

### 2.4. Preparation of Core-Shell Si-ILs Catalyst

The catalyst preparation followed a procedure outlined in the literature [21,24]. Initially, 6.65 mg of 1-Butyl-3-methylimidazolium bromide (BMIMBr, molecular weight 219.12 g/mol), 0.6 mg of hexamethylbenzene (molecular weight 162.27 g/mol), and 2.6 g of KCl were weighed and added to a solution of 30 mL of 2 M HCl. This suspension was stirred for 2 h at room temperature. Subsequently, 2.08 g of tetraethyl orthosilicate (TEOS) was added, and the solution was stirred for 24 h at room temperature. The mixture was then heated under reflux for 24 h at 100 °C. Finally, the solution was filtered and dried overnight at 80 °C. The resulting catalyst contained an ionic liquid to TEOS ratio of 0.32 wt%.

### 2.5. Preparation of Core-Shell Si-ILs-ZnO Catalyst

The preparation of this modified catalyst follows a similar procedure to the previous one. Specifically, 12.8 mg of 1-Butyl-3-methylimidazolium bromide (BmimBr, 0.62 wt% of TEOS), 0.6 g of hexamethylbenzene (HMB), 2.6 g of KCl, and 75 mg of ZnO were added to a 30 mL solution of 2M HCl. The mixture was stirred for 2 h at room temperature. Subsequently, the solution was heated under reflux at 100 °C for 24 h, followed by filtration and overnight drying.

### 2.6. Preparation of Sc(III)silicate (Thortveitite) Catalyst

The synthesis of this catalyst, previously employed in another study of ours [23], involves the combination of two established experimental protocols [25,26]. Scandium was impregnated onto the silica support with a high ScO/mesSiO2 ratio (7 mmol/500 mg) to maximize scandium deposition on the catalyst. For this suspension, 250 mg of KIT-6 was suspended in a solution containing 1.73 g of scandium trifluoromethanesulfonate (Sc(OTf)_3_, 3.5 mmol) and 672 mg of citric acid (C_6_H_8_O_7_, 3.5 mmol) as a chelating agent, and then dissolved in 10 mL of a water/ethanol mixture (1:3 *v*/*v*). The mixture was heated under reflux for 24 h.

In the final phase, the solvent was evaporated at 100 °C for 12 h, and the solid residue was calcined at 700 °C for 5 h. The resulting catalyst was classified as Sc(III)silicate (thortveitite). The silica template was then removed by treatment three times with 2M sodium hydroxide. The newly synthesized catalyst was collected by centrifugation, washed with water and ethanol, and dried at 80 °C, yielding the final product.

### 2.7. Catalytic Test: Depolymerization Reaction

To facilitate the dissolution of polycarbonate (PC) beads and to conduct all associated reactions, methyl tetrahydrofuran (m-THF) was employed as the solvent. m-THF is considered a green solvent in comparison to traditional tetrahydrofuran (THF) due to its lower environmental impact and its recommendation in solvent selection guides, as it can be derived from renewable resources.

The depolymerization reaction employed oxygen-based nucleophiles, specifically phenol and methanol, as well as nitrogen-based nucleophiles like benzylamine and dibutylamine. Catalytic tests were carried out using all prepared catalysts: core-shell Si-ILs, core-shell Si-ILs-ZnO, and Sc(III)silicate (thortveitite).

All reactions were performed in a stainless-steel autoclave equipped with a heating jacket and magnetic stirring system. To avoid direct contact between the reaction mixture and the metal surface of the autoclave, the reactions were conducted inside a beaker placed within the autoclave.

In a typical experimental setup, a 50 mL glass vial was charged with 5 mL of m-THF and 125 mg of bisphenol A-based polycarbonate (BPA-PC). This mixture was stirred vigorously at room temperature for 10 min to ensure complete dissolution of the polymer. After dissolution, 25 mg of catalyst and a defined volume of the nucleophile were introduced (see Table 1). The vial was then placed in a stainless-steel autoclave, which was subsequently sealed and heated to the temperature required for the specific catalytic reaction under investigation. Upon completion of the reaction, the mixture was allowed to cool to room temperature and centrifuged to separate the catalyst and unreacted polycarbonate. The supernatant was collected and diluted to a ratio of 1:10 with m-THF for analysis.

A 2 µL aliquot of the diluted solution was injected into a gas chromatograph-mass spectrometer (GC-MS) to quantify the bisphenol A (BPA) concentration. The depolymerization yield was calculated based on a previously established method, with a capping agent serving as an internal reference [10].

### 2.8. Catalyst Recycling

Upon completion of the reaction, the resulting solution, containing the product, was filtered, and the catalyst residue was washed with water. The catalyst was then dried at 60 °C for 24 h.

### 2.9. Acidity Evaluation of Sc(III)silicate (Thortveitite)

Following a published procedure [27], a sample of 26.0 mg of Si-ScO was accurately weighed and suspended in a 2.5 mL solution of NaCl with a concentration of 200 ppm. The mixture was then stirred at room temperature for 24 h to facilitate the ion exchange of H^+^ ions with Na^+^. After the stirring period, the catalyst was filtered and thoroughly washed with deionized water. The filtrate was subsequently titrated with a 0.01 M NaOH solution, employing phenolphthalein as the pH indicator.

## 3. Results and Discussion

**Figure 2:**
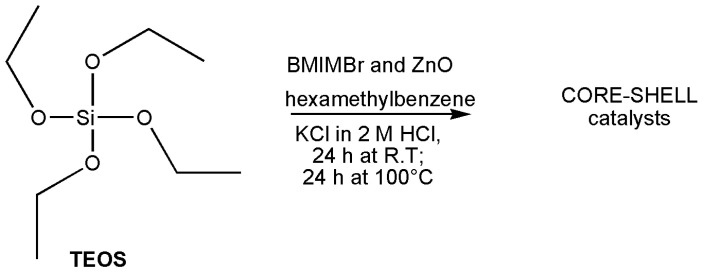
One-step synthesis of BMIMBr confined inside mSiO2 [21].

The catalysts were synthesized according to the procedure used in the literature [21,24], as reported in the experimental section. In this synthesis, the BMIMBr, normally used as a template agent [24], together with hexamethylbenzene, gives the formation of mesoporous silica, enabling pore size regulation based on the IL quantity (Figure 2).

**Figure 3:**
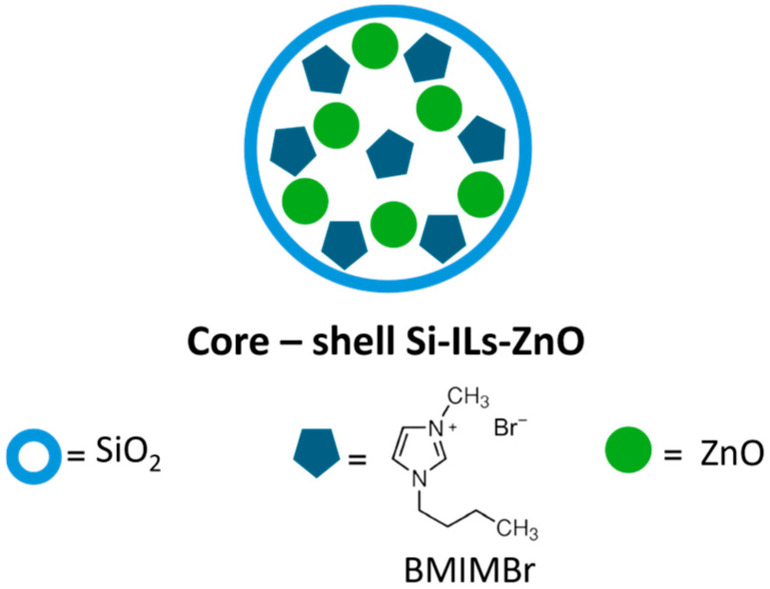
Confinated BMIMBr and ZnO in mesoporous silica gel.

In this case, the ionic liquid (IL) plays a double role, in fact acting as a template during the formation of the mesoporous silica, enabling pore size regulation based on the IL quantity, but also being incorporated during the silica structure formation, contrasting with the conventional method where silica, specifically the Si-OH groups, is functionalized in a subsequent reaction step, following the “one-step assembly” method (Figure 3).

### 3.1. Characterization of Catalysts

#### 3.1.1. X-Ray Powder Diffraction and FE-SEM-EDS Characterization

**Figure 4:**
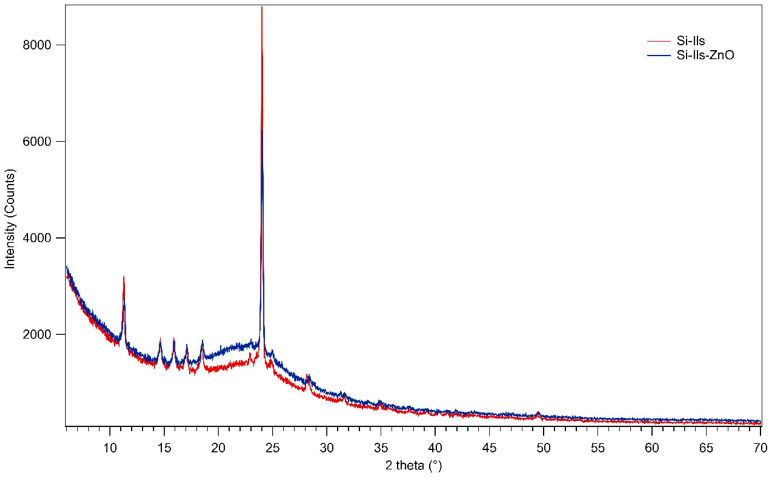
XRPD patterns of core-shell catalysts Si-Ils and Si-Ils-ZnO.

The X-ray powder diffraction (XRPD) pattern showed the occurrence of hexamethylbenzene (C_12_H_18_) as the only crystalline phase (ICDD reference code 33-1695) in both the catalysts, in addition to the typical broad band of amorphous silica centred at about 2θ = 22–23° (Figure 4). The latter appeared more intense in the core-shell Si-Ils-ZnO than in the core-shell Si-Ils catalyst, indicating a higher content of amorphous silica in the former catalyst. Furthermore, the main observed diffraction peak of ZnO overlaps with the reflection at 2θ = 31.82° of hexamethylbenzene, therefore contributing to the difficulty in detecting the occurrence of small ZnO diffraction peaks if very long measuring times are used. To verify the presence of ZnO in the catalyst, an inductively coupled plasma optical emission spectroscopy (ICP-OES) analysis was conducted, following the methodology outlined in the literature [28]. The analysis identified a ZnO concentration of 136 ppm (0.014% in our catalyst), indicating a significant loss of active material during the synthesis, in accordance with the published data [28] (see Supplemental Materials for details of the analysis, specifically Figures S1 and S2).

**Figure 5:**
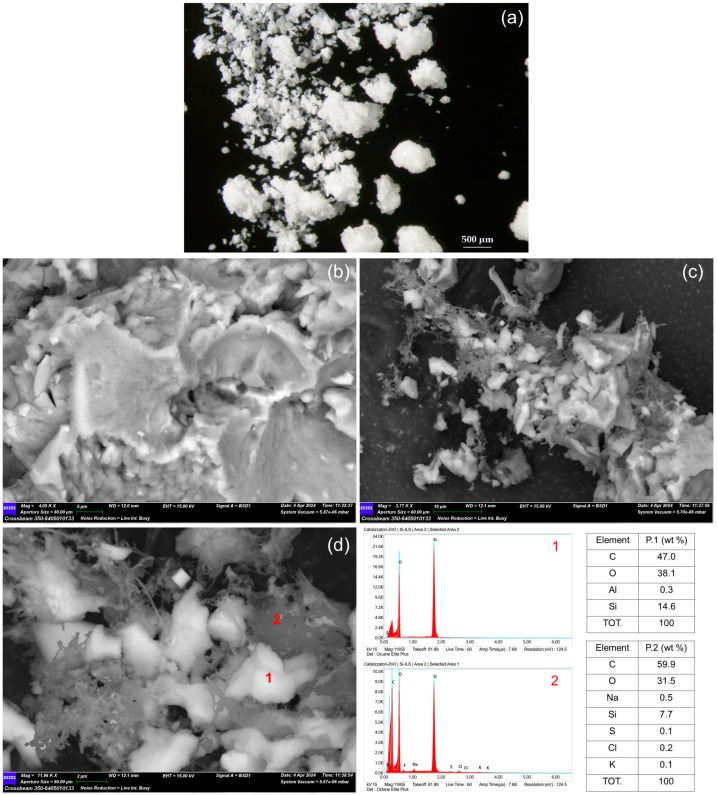
Core-shell catalyst Si-Ils: (**a**) stereomicrocope image (enlargement 20×); (**b**–**d**) SEM images at different magnifications. EDS analyses of points 1 and 2 in graphs and tables on the bottom-right.

**Figure 6:**
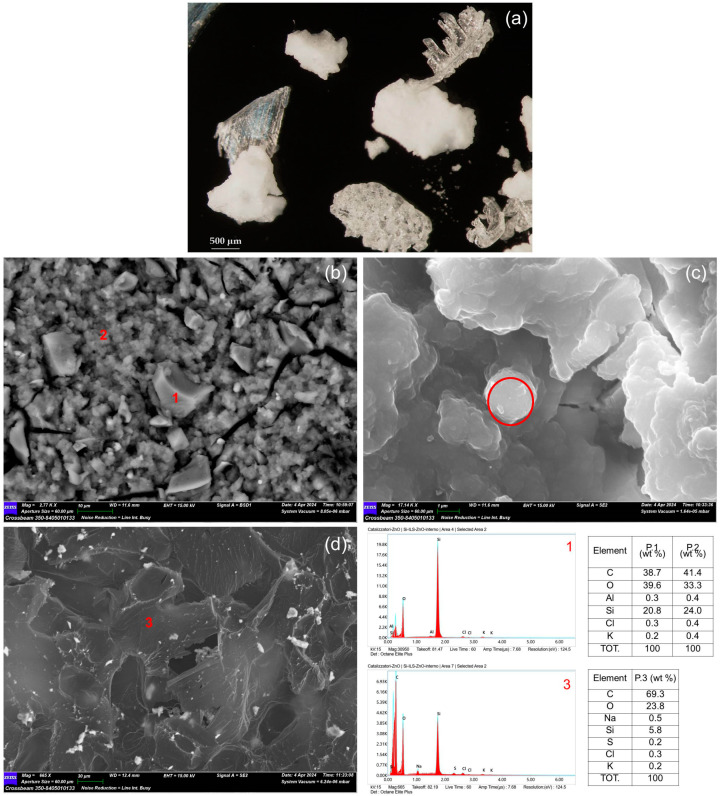
Core-shell catalyst Si-Ils-ZnO: (**a**) stereomicroscope image (enlargement 20×); (**b**–**d**) SEM images at different magnifications. EDS analyses of points 1, 2, and 3, respectively, in graphs and tables on the bottom-right.

Under optical microscopy, the core-shell Si-ILs catalyst appears as homogenous fine particles aggregates of a micrometric size, whereas the core-shell Si-Ils-ZnO catalysts showed a segregation into two components, i.e., compact opaque grains and transparent plates (Figure 5a and Figure 6a). FE-SEM-EDS allowed for a detailed morphological and chemical characterization of both catalysts. High resolution images of the core-shell Si-Ils catalyst showed the occurrence of irregular micrometric silica grains finely intermixed with dendritic particle aggregates (Figure 5c,d). According to the EDS analyses, the former can be attributed to the silica-rich component of the core-shell catalyst and the latter to an organic compound presumably consisting of hexamethylbenzene, whose occurrence was clearly revealed by the XRPD analyses (Figure 4). In contrast, the core-shell Si-ILs-ZnO catalyst is characterized by an almost different morphology, as it consists of compact nanometric grain clusters and some micrometric-sized grains, both showing silicious composition (Figure 6b,c) despite the presence of IL tending to aggregate outside the as-formed mSiO_2_ skeleton. No zinc or nitrogen were detected by the EDS analyses in any portion of the catalyst, as expected. This result confirms that zinc is therefore confined in the catalyst (see also Supplementary Data, Figure S3).

The thin lamella in the SEM image in Figure 6d corresponding to the transparent plates observed through the microscope showed a high amount of carbon, suggesting it to be hexamethylbenzene, in agreement with the XRPD analyses (Figure 4).

**Figure 7:**
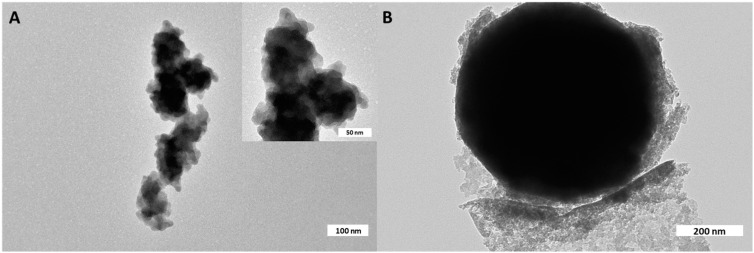
(**A**,**B**) TEM micrographs of Si-Ils-ZnO at different magnifications.

A further investigation to determine the structure of the Si-Ils-ZnO catalyst was carried out using transmission electron microscopy (TEM) analysis (Figure 7).

**Figure 8:**
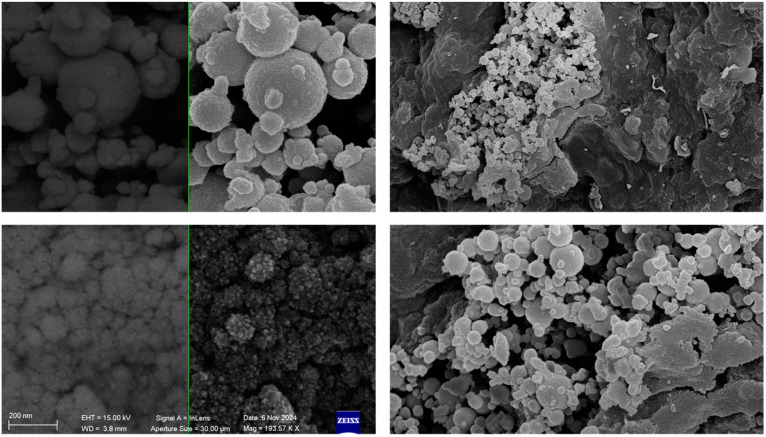
FESEM images of Si-Ils-ZnO Si at different magnifications.

TEM images of the Si-Ils-ZnO catalyst highlight the presence of spherical objects sizing more than 600 nm, which is in good agreement with the spherical objects observed in the SEM images (Figure 8), and smaller nanoparticles (diameter 15 nm) organized in sub-micrometric aggregates of irregular shape [21,29], demonstrating that the structure of the core-shell catalyst was similar to those reported in the literature [28,30].

#### 3.1.2. ATR-IR Analyses

**Figure 9:**
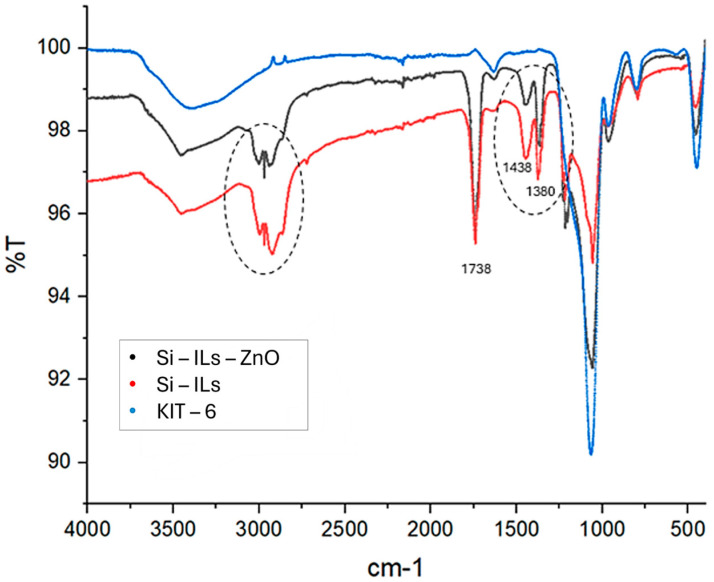
Overlapped ATR spectra of catalysts Si-ILs-ZnO, Si-ILs, KIT-6.

ATR-IR spectra of the KIT-6 catalyst (mesoporous silica), core-shell Si-ILs catalyst, and core-shell Si-Ils-ZnO catalyst were acquired (Figure 9).

In the spectral analysis, distinct signals were observed at 1438 cm^−1^, 1380 cm^−1^, and 1738 cm^−1^, which are characteristic of the imidazole ring. These signals are notably absent in the spectrum of the mesoporous silica (KIT-6). Additionally, the catalysts containing ionic liquid exhibited a group of signals in the range of 2800–3000 cm^−1^, indicative of C-H stretching in the aliphatic chains that is typical of ionic liquids. However, the diagnostic signals for zinc oxide are not discernible, likely due to their overlap with the stretching and bending signals associated with the Si-O bonds in silica, which are Si-O-Si asymmetric stretching around 1080 cm^−1^, Si-O-Si bending near 800 cm^−1^, and Si-O-Si symmetric stretching around 450 cm^−1^.

#### 3.1.3. Thermogravimetric Analysis

**Figure 10:**
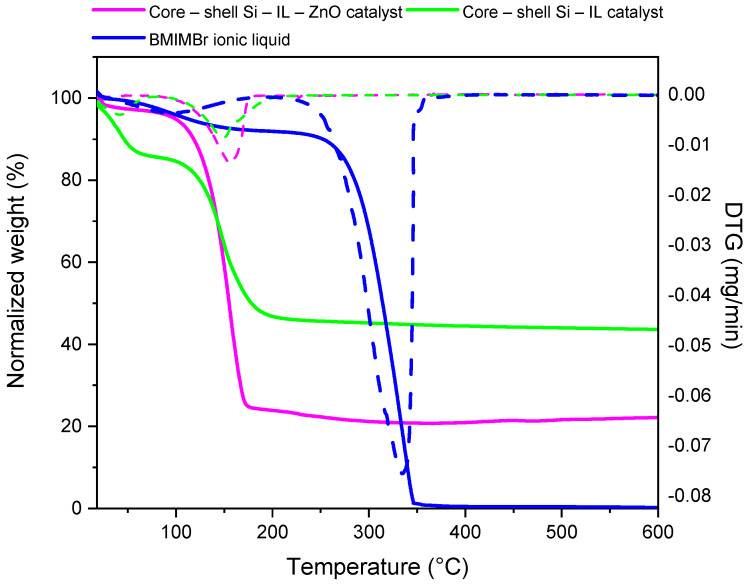
TGA curves (solid lines) and DTG curves (dash lines) of pure BMIMBr ionic liquid (blue line), core-shell Si-IL-ZnO catalyst (purple line), and core-shell Si-IL catalyst (green line).

Thermogravimetric analysis (TGA) was employed to characterize both the core-shell Si-IL catalysts and the core-shell Si-ILs-ZnO catalyst and confirm the presence of the ionic liquid within the silica pores (Figure 10).

The thermogravimetric analysis of the pure ionic liquid BMIMBr was performed first (Figure 10, blue line). It was found that the decomposition process starts around 293 °C and concludes around 350 °C, with the peak temperature being detected at 333 °C according to published data [31,32]. The core-shell catalysts were then characterized (Figure 10, purple and green lines). The data clearly indicate a decrease in the decomposition onset temperature of BMIMBr around 130 °C in both cases, likely due to interactions between the ionic liquid and the nanoscale structure of the catalysts in which it is confined. This observation is consistent with findings reported in the literature [33]. The DTG curves reveal a small weight loss of the ionic liquid, consistent with the small amount used for the preparation of the catalysts.

All the analyses that were carried out and their comparison highlight the success of the core-shell synthesis of the obtained catalysts, which were then tested in the chosen depolymerization reaction.

### 3.2. Catalytic Tests

#### 3.2.1. Depolymerization Reaction Using Sc(III)silicate (Thortveitite) Catalyst

Initially, the Sc(III) silicate catalyst (thortveitite), previously utilized by our research group for other processes, was employed in the depolymerization reaction. Alcohols, specifically phenol and methanol, were used as nucleophiles. Furthermore, we also investigated nitrogen-based nucleophiles, particularly amines, due to their potential to synthesize valuable ureas and their derivatives from waste polycarbonate. The experimental conditions were chosen as follows:

The reaction described in Table 2, Entry 1, returned a yield of 53%. In contrast, when the reaction was performed without a catalyst (Table 2, Entry 3), a reduced yield was observed. The temperature played a crucial role in the process, with a 10 °C decrease leading to a sharp decline in the yield, even lower than that of the non-catalyzed reaction at 150 °C. GC-MS analysis of the product obtained using phenol as the nucleophile revealed the presence of 3-phenoxyphenol, suggesting its formation as a by-product via the dimerization of the phenol. This observation implies competition between the nucleophilic attack on the carbonyl group and the phenol dimerization, which diminishes the overall yield. Due to the acidic nature of phenol (pKa = 10), it likely accelerates the dimerization pathway, further hindering the reaction efficiency. Additionally, the catalyst exhibited acidic properties (0.08 mmol H^+^/g), as determined by the method outlined in the ENEA report [27] that was previously described, which may promote the formation of 3-phenoxyphenol. The acidity of the catalyst indicates that both the catalyst itself and the phenol contribute to weak acid catalysis. In contrast, methanol (pKa = 15.5), being less acidic but a more effective nucleophile, led to a significant improvement in the yield, showing a three-fold increase when comparing the catalysed reaction (Table 2, Entry 4) to that of the methanol blank (Table 2, Entry 5). To overcome the autocatalytic effect observed with phenol, nitrogen nucleophiles were tested as alternatives. The use of primary (benzylamine) and secondary amines (dibutylamine) (Table 2, Entries 6, 7, and 8) resulted in a marked increase in the yield, demonstrating the effectiveness of nitrogen nucleophiles in enhancing the reaction’s efficiency [34]. For comparative analysis, the results of the methanolysis reaction of polycarbonate with an organocatalyst, specifically 1,5,7-triazabicyclo [4.4.0]dec-5-ene (TBD), under both homogeneous and heterogeneous conditions are reported. These results demonstrate that scandium, in this context, is not the most effective catalyst for achieving high-efficiency depolymerization (Table 2, Entries 8 and 9).

#### 3.2.2. Depolymerization of Polycarbonate Employing Core-Shell Catalyst (Si-ILs/Si-ILs-ZnO)

The potential of a core-shell catalyst, utilizing BMIMBr as the ionic liquid, was investigated. The depolymerization reactions of this catalyst in two forms were tested: as a Si-IL shell catalyst and with the addition of a metal oxide, specifically ZnO. Zinc-based oxides are preferred due to zinc’s exclusion from the European Union’s list of critical raw materials, which identifies substances of significant economic importance for the EU’s supply chains. In contrast to zinc, scandium has recently been added to this list. The reaction was initiated under optimal conditions that were previously determined for the Sc(III)silicate (thortveitite) catalyst. We chose methanol as a nucleophile due to its utility in producing dimethyl carbonate, and we also tested benzylamine, which yielded the highest results (see Table 3, Entry 8). The reaction was conducted at 150 °C for a duration of 12 h. Consistent quantities of the various reagents were employed in all reactions, facilitating a comparative analysis between the Si-ILs and Si-ILs-ZnO.

The optimal reaction conditions for the Si-IL catalysts are outlined in Table 3, Entry 2. To investigate the possibility of conducting the reaction under milder conditions, the reaction temperature was lowered to 90 °C (Table 3, Entry 3). This temperature reduction led to a substantial drop in the yield to 13%, with unreacted polycarbonate (PC) beads accumulating at the bottom of the beaker. Further experiments utilized the Si-ILs-ZnO catalyst, which has a surface acidity of 0.04 mmol H+/g, indicating that it is less acidic than the Sc(III)silicate (thortveitite) catalyst. As seen in Entries 4 and 5, the presence of the Si-ILs-ZnO catalyst significantly improved the yield compared to the non-catalyzed reaction (Table 2, entry 8), in which the yield was negligible and unquantifiable. In addition, the reaction detailed in Table 3, Entry 1 showed a yield of 49%, lower than that of the reaction carried out with Si-ILs-ZnO in the same reaction conditions (Table 3, entry 5, 75%).

Under the same conditions as the reaction shown in Table 3, Entry 5, the use of different nucleophiles (Entries 6 and 7) showed consistent behavior with that of the Sc(III)silicate (thortveitite) catalyst, with nitrogen nucleophiles yielding higher results than oxygen nucleophiles. Moreover, when methanol is selected as a nucleophile and the conditions of reaction are fixed, the yield of the reaction remains the same regardless of the presence of ZnO. This can be explained considering that the acidic properties of methanol outweigh the catalytic properties of ZnO (Entries 2 and 6).

An analysis of the effect of temperature (Entries 7–10) demonstrated that, while lower temperatures reduced the yield, the impact was not drastic. Even at 60 °C, considered a relatively mild condition, the yield remained close to 60%. Additionally, varying the reaction time between 12 and 24 h (Entry 8) had no significant effect on the yield, indicating that the temperature, rather than the reaction duration, is the key factor influencing the reaction’s efficiency. The comparison with data from the literature (Table 3, Entries 12 and 13) showed that it is possible to obtain similar yields while adding the undoubted advantages of using a heterogeneous catalyst, which can be easily recovered and reused for at least five subsequent cycles without a loss of reactivity.

All these data showed that the confinement effect allows using the minimum quantity of metal and ionic liquid, in accordance with economic and energy saving criteria [21]. In addition, the Si-ILs-ZnO catalyst represents, to our knowledge [13,34], the first heterogeneous catalyst that is able to obtain the ammonolysis of polycarbonates, opening the way for the reuse of urea derivatives.

#### 3.2.3. Gel Permeation Chromatography Analysis of the Depolymerization Product

To determine the size of the remaining polymer chains, gel permeation chromatography (GPC) was performed. The solution containing the product of the reaction listed in Table 4, Entries 1, 2 and 3 were diluted to a maximum concentration of 1 mg/mL to prevent clogging of the chromatographic column.

**Figure 11:**
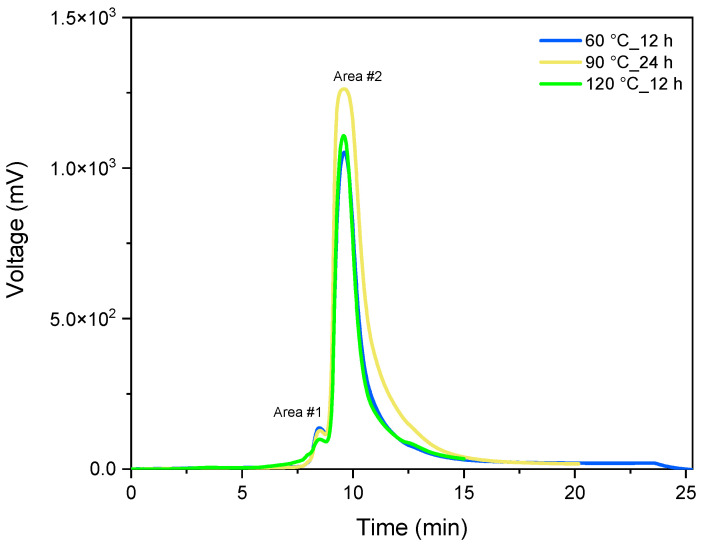
Overlay of the GPC chromatograms obtained from the three reactions listed in the following table, Table 4.

The chromatogram analysis (Figure 11) shows a consistent fragmentation pattern of the initial polymer chain across all samples, as evidenced by the two distinct peaks. The first peak, appearing around 8 min, corresponds to a nominal mass of approximately 15,000 g/mol. Since the starting polymer chain has a nominal mass of 45,000 g/mol, this suggests that the chain has broken into oligomers about one-third the size of the original polymer. A second, broader peak emerges around 9 min, slightly overlapping with the calibration curve. Only the portion of the peak that lies within the calibration range was considered, yielding an estimated value of 1500 g/mol. This points to substantial depolymerization of the polycarbonate into much smaller units, as the base unit mass is 256.11 g/mol. The downward slope of the peak toward lower values indicates the possible presence of monomeric or dimeric units.

A qualitative evaluation of the reaction progress can be conducted by examining the peak areas shown in Table 4.

Specifically, it is observed that an extended reaction time results in a peak that is approximately 50% higher. This phenomenon can be explained by hypothesizing that longer reaction durations lead to increased fragmentation of the polymer chain, yielding lower mass oligomers (approximately 1500 g/mol) rather than individual monomer units. In contrast, conducting the reaction at 120 °C for 12 h results in a higher yield of monomer units, thus producing a peak that skews towards lower molecular weights.

#### 3.2.4. First Application: Depolymerization of a DVD-R

**Figure 12:**
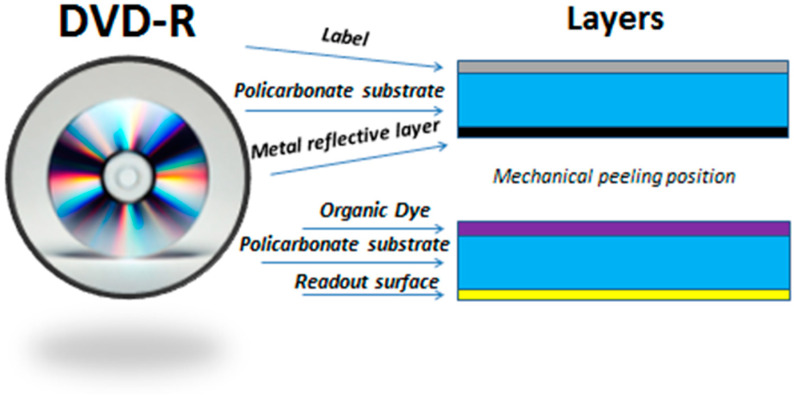
DVD-R structure.

Based on the results obtained from a sample of pure polycarbonate (Sigma-Aldrich), we attempted to apply the reaction to a real-world sample under optimal conditions, specifically to a DVD-R. The structure of a DVD consists of several layers, as depicted in Figure 12 [36]. These layers include a printable surface, a polycarbonate substrate, a reflective metal layer, an organic dye, an organic substrate, and a reading surface. Given that the primary component of a DVD is polycarbonate, they serve as an appropriate real-world sample for the application of the reaction developed on a laboratory scale.

In the study conducted by Alberti et al. [37], the substrate underwent no pretreatment; instead, a depolymerization reaction was performed directly on the sample. Building on this approach, the most promising depolymerization reaction identified in the laboratory was applied to a DVD-R. The DVD-R was first appropriately chopped, and then the reaction was conducted for 12 h at 120 °C using 125 mg of DVD-R, 5 mL of m-THF, 1 mL of benzylamine, and 25 mg of Si-ILs-ZnO catalyst.

After filtering the sample to remove all impurities, the yield was calculated to be 75%. However, it may be necessary to optimize the reaction by pre-treating the DVD-R to achieve a higher yield.

#### 3.2.5. Second Application: Depolymerization of Polyol-Polyester

Depolymerization was attempted using a polyol-polyester and a polyurethane synthesized in our previous work [23]. A polyol-polyester derived from succinic acid and glycerol (Mn distribution shown in Table 5) was treated with 25 mg of Si-ILs-ZnO catalyst, using 1 mL of methanol as the nucleophile and 5 mL of THF as the solvent, for 12 h at 120 °C.

Upon completion of the reaction, no solid residue remained. GC analyses revealed peaks corresponding to glycerol and succinic acid, indicating that complete depolymerization of the polyol-polyester had occurred. GPC analyses showed no traces of residual polymers.

#### 3.2.6. Third Application: Depolymerization of Polyurethane

In our previous work [23], a polyurethane was synthesized from the reaction between the polyol-polyester described in the previous section and Wannate HT 100 Plus (1,6-hexamethylene diisocyanate, oligomers). The polyurethane was then treated with 25 mg of Si-ILs-ZnO catalyst, using 1 mL of methanol as the nucleophile and 5 mL of methyl-THF as the solvent, for 12 h at 120 °C. Upon completion of the reaction, no solid residue remained. GC analyses revealed peaks corresponding to glycerol and succinic acid (derived from the polyol-polyester component of the polymer), indicating complete depolymerization of the polyurethane. GPC analyses showed no traces of residual polymers.

## 4. Conclusions

Based on the collected data, the Si-ILs-ZnO shell catalyst studied herein demonstrates high efficiency in the depolymerization process, achieving a bisphenol A (BPA) yield of 76% at 120 °C. Furthermore, it has been shown that, operating under milder conditions, specifically at 60 °C, the yield is approximately 60%. The depolymerization process facilitates the cleavage of bonds within the polymer chain, predominantly resulting in the formation of monomeric units that are viable for new polycarbonate production. Additionally, the residual polycarbonate chain primarily consists of oligomers or polymer chains that are shorter than the original polycarbonate chain. These findings are particularly significant as they introduce a method for the chemical recycling of polycarbonate using a novel catalyst based on ionic liquids.

Based on the results obtained herein, we conclude that a novel core-shell catalyst has been developed, offering several advantages:

- The catalyst facilitates the ammonolysis of polycarbonates, enabling the reuse of urea derivatives;
- It is also effective for the depolymerization of various condensation polymers, including polyesters and polyurethanes;
- The catalyst has demonstrated exceptional robustness, maintaining activity through at least five recycling cycles.

These findings indicate significant potential for further investigation into additional polymer categories to expand the applicability of the catalyst.

## Figures and Tables

**Table 1:** Summary of nucleophiles and catalysts employed in catalytic tests ^a^.

Nucleophile	Catalyst ^b^
Phenol	
Methanol	Core-shell Si-ILs
Dibutylamine	Core-shell Si-ILs-ZnO
Benzylamine	Sc(III)silicate (thortveitite)

^a^: for each reaction, m-THF was used as solvent (5 mL) and PC as substrate (125 mg). ^b^: for each reaction, 25 mg of catalyst was used.

**Table 2:** Depolymerization reaction using Sc(III)silicate (thortveitite) catalyst (^a^) compared with processes in the literature.

Entry	Nucleophile ^b^	T (°C)	Yield (%)	Catalyst	Reference #
1	Phenol	150	53	^a^	This work
2	Phenol	140	8	^a^	This work
3 ^c^	Phenol	150	35	^a^	This work
4	Methanol	150	29	^a^	This work
5 ^c^	Methanol	150	10	^a^	This work
6	Dibutylamine	150	52	^a^	This work
7	Benzylamine	150	58	^a^	This work
8 ^c^	Benzylamine	150	Traces	^a^	This work
9 ^d^	Methanol	RT	88	TBD	[7]
10 ^e^	Methanol	65	>90	Si-TBD	[8]

^a^: for each reaction, m-THF was used as solvent (5 mL), Sc(III)silicate (thortveitite) as catalyst (25 mg), and PC as substrate (125 mg), time: (h) 12 h. ^b^: reaction with methanol (Entries 4 and 5) carried out using 0.5 mL of nucleophile. All the other reactions were carried out with 1 mL of nucleophile. ^c^: carried out without catalyst (blank). ^d^: homogeneous catalysis. ^e^: heterogeneous catalysis.

**Table 3:** Depolymerization reaction using Si-ILs/Si-ILs-ZnO catalysts and benzylamine/methanol as nucleophile ^a^.

Entry	Catalyst ^b^	Nucleophile ^c^	T (°C)	Time (h)	Yield ^e^ (%)	Ref.
1	SI-ILs	Benzylamine	150	12	49	This work
2	Si-ILs	Methanol	150	12	60	This work
3	Si-ILs	Methanol	90	12	13	This work
4 ^d^	SI-ILs-ZnO	Benzylamine	150	12	Traces	This work
5	Si-ILs-ZnO	Benzylamine	150	12	75	This work
6	Si-ILs-ZnO	Methanol	150	12	60	This work
7	Si-ILs-ZnO	Benzylamine	120	12	76	This work
8	Si-ILs-ZnO	Benzylamine	90	24	57	This work
9	Si-ILs-ZnO	Benzylamine	90	12	59	This work
10	Si-ILs-ZnO	Benzylamine	60	12	58	This work
11 ^f^	-----	Benzylamine	80	12	63	[35]
12 ^g^	NBu_4_Cl/ZnO-NP	Methanol	100	7	>90	[10]
13 ^g^	NBu_4_Cl/ZnO-NP	Cyclohexylamine	100	7	90	[10]

^a^: for each reaction, m-THF was used as solvent (5 mL) and PC as substrate (125 mg). ^b^: for each reaction, 25 mg of catalyst was used. ^c^: for each reaction, 1 mL of nucleophile was used. ^d^: carried out without solvent. ^e^: all yield values were obtained based on three replicate experiments (SD ± 3). ^f^: i-PrOH solvent. ^g^: N_2_, atmospheric pressure.

**Table 4:** List of samples analysed with GPC ^a^.

Entry	T (°C)	Time (h)	Area #1	Area #2	Yield (%)
1	120	12	1970	86,687	76
2	90	24	4711	92,299	57
3	60	12	934	144,938	58

^a^: for each reaction, Si-ILs-ZnO was used as catalyst (25 mg), PC as substrate (125 mg), m-THF as solvent (5 mL), and benzylamine as nucleophile (1 mL).

**Table 5:** Mn distribution of the polyol-polyester.

M_n1_ (Da) (%)	M_n2_ (Da) (%)	M_n3_ (Da) (%)	M_n4_ (Da) (%)
26,664 (4.53)	12,100 (15.76)	3198 (18.46)	1633 (61.32)

## Data Availability

All the research data were reported in the paper.

## Supplementary Materials

The following supporting information can be downloaded at: https://www.mdpi.com/article/10.3390/polym16223209/s1, Figure S1: Calibration curve for ICP-OES analysis and results for the catalyst (line 43); Figure S2: Graphic for the sample Si-Ils ZnO (line 43); Figure S3: Energy Dispersive Spectroscopy (EDS) analysis of Core–shell Si-ILs-ZnO catalyst.
