# Supplementary material for: Demonstrating the Efficacy of Core-Shell Silica Catalyst in Depolymerizing Polycarbonate
Source: Polymers (Basel). 2024 Nov 19;16(22):3209. doi: 10.3390/polym16223209 (PMC11598560; doi:10.3390/polym16223209)
Supplement: Supplementary file 1 [file polymers-16-03209-s001.zip › polymers-3278224-supplementary.pdf]

Supplementary data

Demonstrating the Efficacy of Core-Shell Silica Catalyst in Depolymerizing Polycarbonate

Onofrio Losito [1], Pasquale Pisani [1], Alessia De Cataldo [1,2], Cosimo Annese [3,5], Marina Clausi [4], Roberto Comparelli[6], Daniela Pinto [4] and Lucia D'Accolti [1,*]

1. Dipartimento di Chimica, Università degli Studi di Bari Aldo Moro, via E. Orabona 4, 70126 Bari, Italy
2. Dipartimento di Meccanica, Matematica e Management (DMMM), Politecnico di Bari, via E. Orabona 4, 70126 Bari, Italy
3. Dipartimento di Scienze della Vita, della Salute e delle Professioni Sanitarie, Università degli Studi Link, via del Casale di San Pio V, 44 00165 Roma – Italy
4 Dipartimento di Scienze della Terra e Geoambientali, Università degli Studi di Bari Aldo Moro, via E. Orabona 4, 70126 Bari, Italy
5. CNR-ICCOM-SS BARI (I) via orabona, 4 70125 Bari (Italy)
6. CNR-IPCF-SS BARI (I) via orabona, 4 70125 Bari (Italy)
* Correspondence: lucia.daccolti@uniba.it

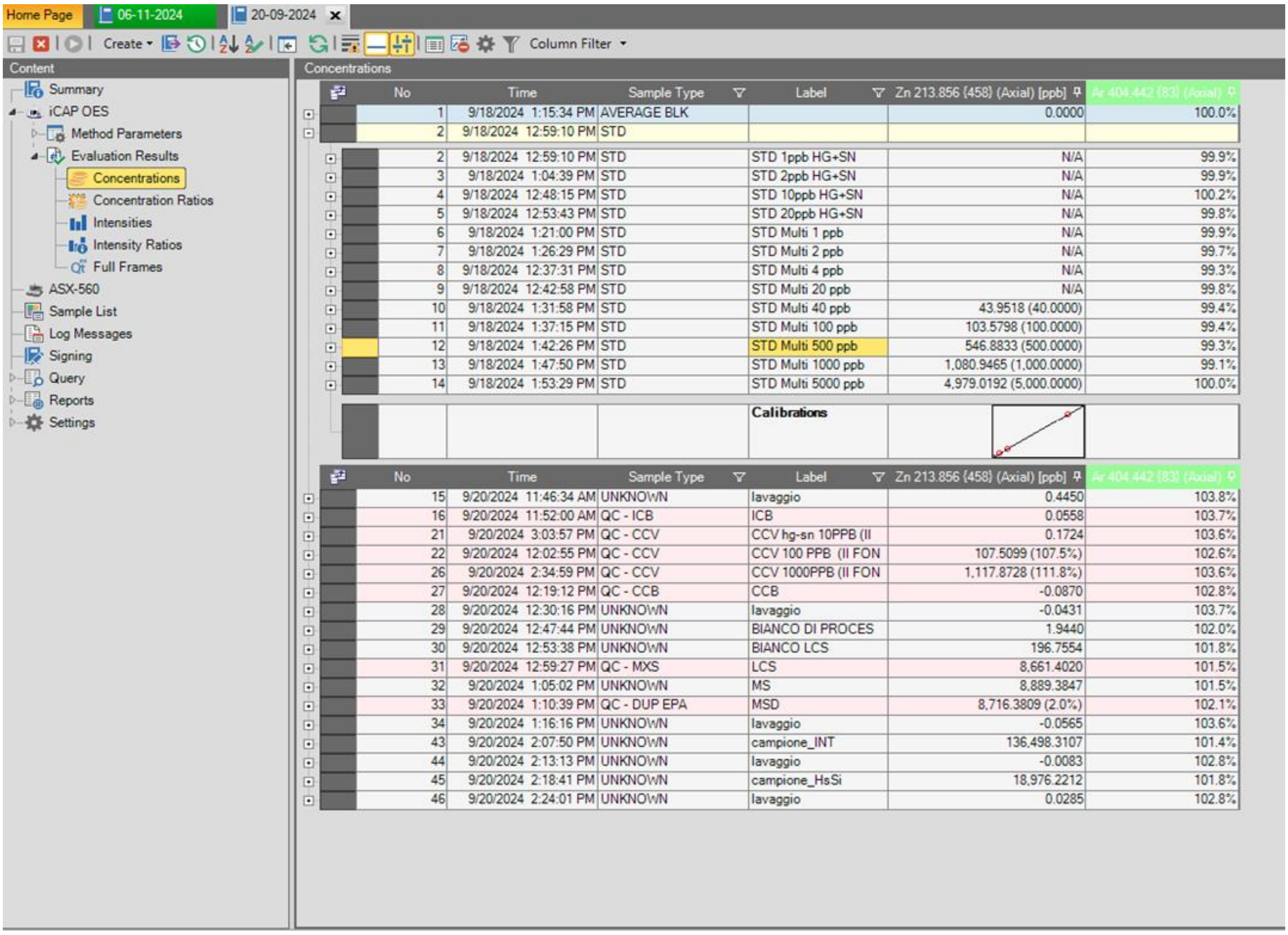

No	Time	Sample Type	Label	Zn 213.856 {458} (Axial) [ppb]	Ar 404.442 {83} (Axial)
1	9/18/2024 1:15:34 PM	AVERAGE BLK		0.0000	100.0%
2	9/18/2024 12:59:10 PM	STD			
2	9/18/2024 12:59:10 PM	STD	STD 1ppb HG+SN	N/A	99.9%
3	9/18/2024 1:04:39 PM	STD	STD 2ppb HG+SN	N/A	99.9%
4	9/18/2024 12:48:15 PM	STD	STD 10ppb HG+SN	N/A	100.2%
5	9/18/2024 12:53:43 PM	STD	STD 20ppb HG+SN	N/A	99.8%
6	9/18/2024 1:21:00 PM	STD	STD Multi 1 ppb	N/A	99.9%
7	9/18/2024 1:26:29 PM	STD	STD Multi 2 ppb	N/A	99.7%
8	9/18/2024 12:37:31 PM	STD	STD Multi 4 ppb	N/A	99.3%
9	9/18/2024 12:42:58 PM	STD	STD Multi 20 ppb	N/A	99.8%
10	9/18/2024 1:31:58 PM	STD	STD Multi 40 ppb	43.9518 (40.0000)	99.4%
11	9/18/2024 1:37:15 PM	STD	STD Multi 100 ppb	103.5798 (100.0000)	99.4%
12	9/18/2024 1:42:26 PM	STD	STD Multi 500 ppb	546.8833 (500.0000)	99.3%
13	9/18/2024 1:47:50 PM	STD	STD Multi 1000 ppb	1,080.9465 (1,000.0000)	99.1%
14	9/18/2024 1:53:29 PM	STD	STD Multi 5000 ppb	4,979.0192 (5,000.0000)	100.0%
			Calibrations		
15	9/20/2024 11:46:34 AM	UNKNOWN	lavaggio	0.4450	103.8%
16	9/20/2024 11:52:00 AM	QC - ICB	ICB	0.0558	103.7%
21	9/20/2024 3:03:57 PM	QC - CCV	CCV hg-sn 10PPB (II	0.1724	103.6%
22	9/20/2024 12:02:55 PM	QC - CCV	CCV 100 PPB (II FON	107.5099 (107.5%)	102.6%
26	9/20/2024 2:34:59 PM	QC - CCV	CCV 1000PPB (II FON	1,117.8728 (111.8%)	103.6%
27	9/20/2024 12:19:12 PM	QC - CCB	CCB	-0.0870	102.8%
28	9/20/2024 12:30:16 PM	UNKNOWN	lavaggio	-0.0431	103.7%
29	9/20/2024 12:47:44 PM	UNKNOWN	BIANCO DI PROCES	1.9440	102.0%
30	9/20/2024 12:53:38 PM	UNKNOWN	BIANCO LCS	196.7554	101.8%
31	9/20/2024 12:59:27 PM	QC - MXS	LCS	8,661.4020	101.5%
32	9/20/2024 1:05:02 PM	UNKNOWN	MS	8,889.3847	101.5%
33	9/20/2024 1:10:39 PM	QC - DUP EPA	MSD	8,716.3809 (2.0%)	102.1%
34	9/20/2024 1:16:16 PM	UNKNOWN	lavaggio	-0.0565	103.6%
43	9/20/2024 2:07:50 PM	UNKNOWN	campione_INT	136,498.3107	101.4%
44	9/20/2024 2:13:13 PM	UNKNOWN	lavaggio	-0.0083	102.8%
45	9/20/2024 2:18:41 PM	UNKNOWN	campione_HsSi	18,976.2212	101.8%
46	9/20/2024 2:24:01 PM	UNKNOWN	lavaggio	0.0285	102.8%

Figure S1. Calibration curve for ICP-OES analysis and results for the catalyst (line 43).

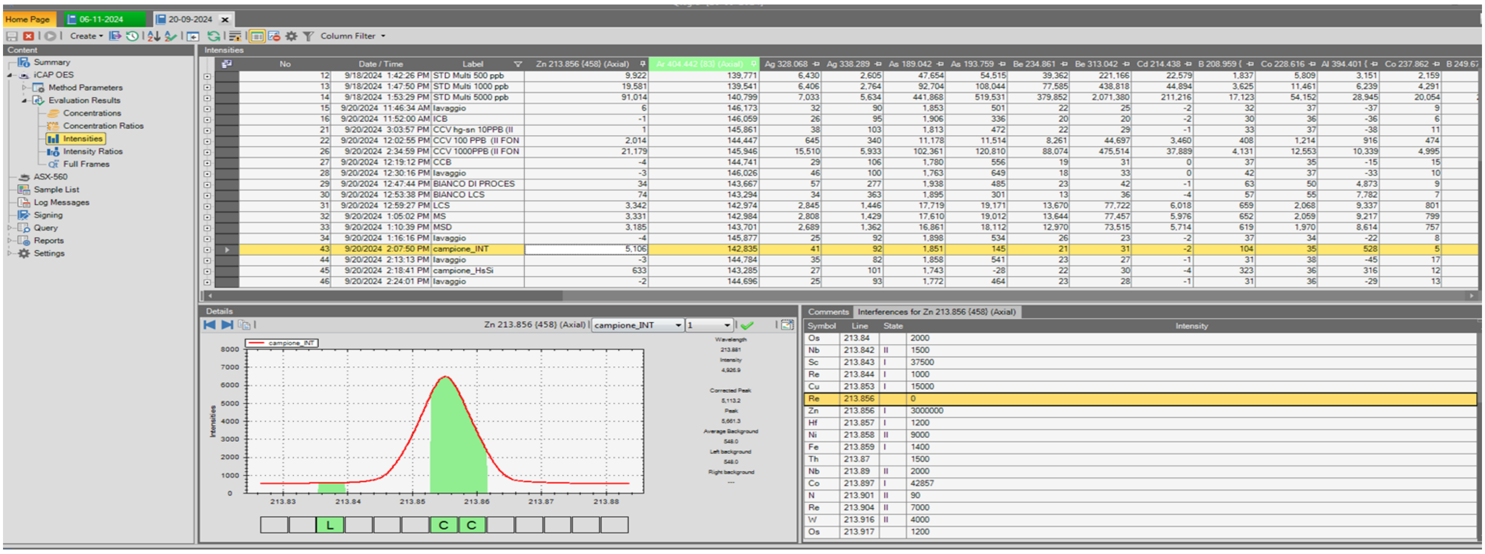

Figure S2 Graphic for the sample Si-Ils ZnO (line 43).

Table S1. eZAF Oxides Quant Result - Analysis Uncertainty: 22.36 %

Element	Weight %	MDL	Atomic %	Error %	Net Int.	R	A	F
O	0.0	0	0.0	16.7	768.6	0.0000	0.0000	0.0000
Si O2	98.2	0.04	97.9	3.6	2682.9	0.9398	0.8011	1.0025
Cl	0.9	0.12	1.4	12.5	28.9	0.9496	0.7893	1.0074
K 2O	0.9	0.13	0.6	17.8	21.0	0.9556	0.8852	1.0124
Zn O	0.1	0.70	0.0	100.0	0.2	0.9864	0.9946	1.1648

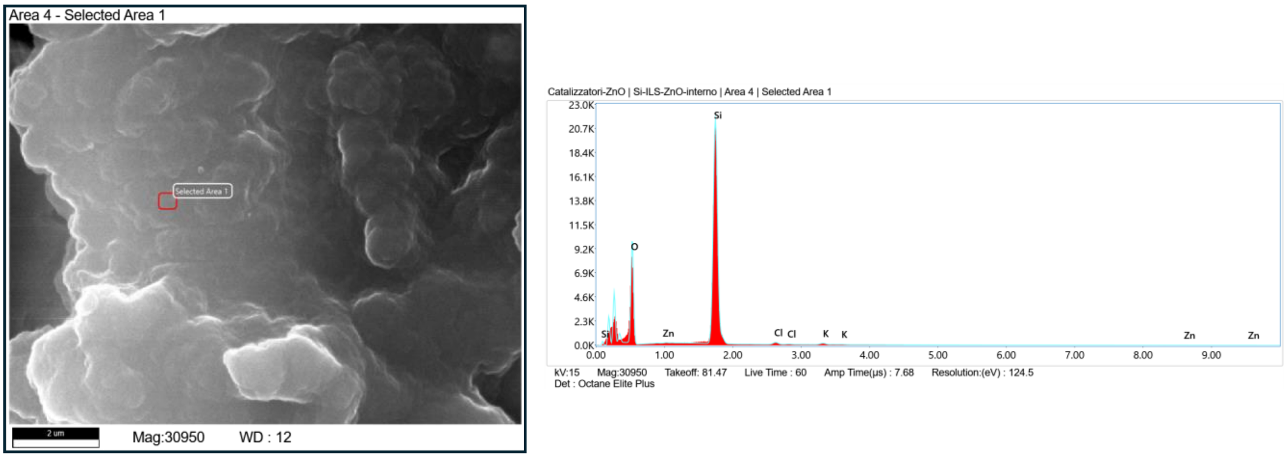

Figure S3. Energy Dispersive Spectroscopy (EDS) analysis of Core – shell Si-ILs-ZnO catalyst.
